# Biochemical and molecular characterization of adult patients with type I Gaucher disease and carrier frequency analysis of Leu444Pro - a common Gaucher disease mutation in India

**DOI:** 10.1186/s12881-018-0687-5

**Published:** 2018-10-01

**Authors:** Jayesh Sheth, Dhairya Pancholi, Mehul Mistri, Payal Nath, Chitra Ankleshwaria, Riddhi Bhavsar, Ratna Puri, Shubha Phadke, Frenny Sheth

**Affiliations:** 1 FRIGE’s Institute of Human Genetics, FRIGE House, Jodhpur Gam Road, Satellite, Ahmedabad, Gujarat 380015 India; 2 Center of Medical Genetics, Sir Ganga Ram Hospital, Rajinder Nagar, New Delhi, 110060 India; 3 Sanjay Gandhi Post Graduate Institute of Medical Science, Lucknow, Uttar Pradesh 226014 India

**Keywords:** Adult Gaucher disease, β-Glucosidase, Chitotriosidase, *GBA* gene, Glucocerebrosidase, Indian population, Leu444Pro carrier frequency

## Abstract

**Background:**

Gaucher disease is a rare pan-ethnic disorder which occurs due to an increased accumulation of undegraded glycolipid glucocerebroside inside the cells’ lysosomes. A beta-Glucosidase (*GBA)* gene defect results in glucocerebrosidase enzyme deficiency. Though the disease is mainly diagnosed in childhood, the adult manifestation is often missed or identified late due to the failure to recognize the heterogeneous clinical presentation. The present study includes seven unrelated Indian adult patients (age range: 20–40 years) having splenomegaly, with or without hepatomegaly, cytopenia and bone abnormality.

**Methods:**

The biochemical investigation implicated measuring plasma chitotriosidase enzyme activity followed by confirmatory test of β-Glucosidase enzyme activity from the leukocytes. The molecular characterization involved patients’ initial screening for the common Gaucher mutation (Leu444Pro). Later, all patients were subjected to whole *GBA* gene coding region study using bidirectional Sanger sequencing. The population screening for common Gaucher disease mutation (Leu444Pro) was executed in 1200 unrelated and healthy Indian subjects by Restriction Fragment Length Polymorphism-Polymerase Chain Reaction technique. The allele frequency was calculated using Hardy-Weinberg formula.

**Results:**

The biochemical analysis revealed a significant reduction in the β-Glucosidase activity in all the patients. Also, an elevated level of plasma Chitotriosidase activity in five patients supported their diagnosis of Gaucher disease. Sanger sequencing established four patients with homozygous variation and three patients with compound heterozygous variation in *GBA* gene. This study uncovers two missense variants (Ala448Thr and Val17Gly) not previously reported in Gaucher disease patients. Also the known mutations like Leu444Pro, Arg329Cys, Asp315Asn, Ser125Arg, and Arg395Cys were identified in these patients. The homology modeling suggested the destabilization of the protein structure due to novel variants. The Leu444Pro mutation screening in the Indian population spotted two people as a carrier. This emerged the carrier frequency of 1:600 along with wild-type allele frequency 0.97113 and mutant allele frequency 0.02887.

**Conclusions:**

The study reports novel and known variants identified in the *GBA* gene in seven adult patients. The given study is the first report on the carrier frequency of the Leu444Pro mutant allele in an Indian population which will help understanding the burden and susceptibility of Gaucher disease to affect next generation in India.

**Electronic supplementary material:**

The online version of this article (10.1186/s12881-018-0687-5) contains supplementary material, which is available to authorized users.

## Background

Gaucher disease (GD, OMIM#230800) is a rare autosomal recessive Lysosomal storage disorder (LSDs) which occurs due to an increased accumulation of undegraded glycolipid glucocerebroside in lysosomes [1]. Lysosomes maintain the cells’ homeostasis by mediating multiple functions like macromolecules breakdown, phagocytosis, and antigen presentation [2]. Several catabolic enzymes degrade macromolecules in Lysosome and make it available to the cell. A mutation in the genes encoding such Lysosomal enzymes either causes loss of function or decreases the functional expression of these enzymes. Such genetic defect results in substrate accumulate in their respective organs and bloodstreams causing various disorders which are broadly categorized as LSDs [3]. Approximately, LSDs covers 50 rare genetic disorders. Our previous study represents that GD is the most common LSD observed in Indian population [4].

Under normal condition, a lysosomal enzyme β-glucosidase (EC 3.2.1.45) catabolize glucocerebroside to glucose and ceramide [5]. Glucosylceramidase beta gene (*GBA*; OMIM*606463), located at the locus 1q22, encodes this enzyme. The loss-of-function mutations in *GBA* gene prevent the enzyme from cleaving the β-glucosyl linkage of Glucocerebroside leading to its accumulation in the lysosomes [6]. Macrophages, involved in eliminating leukocytes containing excess glycosphingolipids, are altered in GD. Such lipid burdened macrophages, under light microscopy, looks enlarged due to eccentric nuclei, and condensation of chromatin and cytoplasm [7]. These cells are called ‘Gaucher cells’ named after Philippe Gaucher who first described it in 1882 in a patient with massive Splenomegaly [8].

Based on the neurological involvement, the GD is classified in three types. Type 1 GD (non-neuronopathic), type 2 GD (acute neuronopathic) and type 3 GD (chronic neuronopathic). Though type 1 GD is heterogenic, the clinical presentations like hepatosplenomegaly, bone disease, and hematological abnormalities like cytopenias are commonly observed [9]. A majority of adult type 1 GD patients are reported in Gaucher registry involving several ethnic groups and races [10, 11]. Few cases of adult type 1 GD are identified from India [12, 13].

Though considered rare, GD is found to be the most common LSD. Its prevalence in general population is estimated to be 1:50000 however, Ashkenazi Jewish population is at higher risk with a prevalence of 1:950 live births [14, 15]. More than 300 pathogenic mutations have now been identified in *GBA* gene but the most commonly occurring mutations like c.1226A > G (Asn370Ser), c.1448T>C (Leu444Pro), c.84dupG (84GG), and c.115 + 1G > A (IVS2 + 1G > A) forms approximately 90% of the total Ashkenazi Jewish GD patients and 50–60% of the total non-Jewish GD patients [16, 17]. The mutation c.1448T>C (Leu444Pro) is pan-ethnic. Our earlier study reported that 60.6% of non-neuronopathic and 3.03% of sub-acute neuronopathic Indian GD patients reported were identified with c.1448T>C (Leu444Pro) mutation [18].

The given study reports seven patients with Type 1 GD manifested in adulthood along with identification of two novel variants in *GBA* gene. The study also demonstrates the carrier frequency analysis of the most common GD mutation (Leu444Pro) identified in an Indian population.

## Methods

### Patients

The present study comprises the patients from clinical cases referred from Institute of Human Genetics after genetic counseling as well as from outside referring physicians. The Ethics committee of the Foundation for Research in Genetics and Endocrinology (FRIGE) at the Institute of Human Genetics approved the study and it was performed in accordance with the tenets of the Declaration of Helsinki. Irrespective of the case reference, a written informed consent for investigation and publication of the data was obtained from the patients or their guardian as per the institutional ethics committee guidelines. The 7 unrelated patients reported, comprises three males and four females in the age range of 20 years to 40 years at the time of investigations. They were referred in the time from 2012 to 2016 with a clinical suspicion of adult GD. The clinical presentation included mild to severe liver/spleen enlargement, anemia, thrombocytopenia and presence of Gaucher cells in bone marrow.

### Biochemical investigations

Six milliliters of blood, drawn from each patient in ethylenediaminetetraacetic acid vacutainer, was subjected to a plasma, leukocyte, and genomic DNA (gDNA) isolation.

### Plasma chitotriosidase screening

The chitotriosidase enzyme activity was measured in blood plasma using a previously described protocol [19]. In brief, the plasma mixed with 4-MU (4-methylumbeliferryl b-D-N,N,N′,N″- triacetylchitotrioside) substrate was incubated at 37 °C. Photo fluorometer with 360 nm primary and 465 nm secondary filter measured the fluorescence.

### Leukocyte enzyme assay

Fluorimetric enzyme assay using 4-methylumbelliferyl-β-D-glucopyranoside substrate measured the Lysosomal hydrolase enzyme (β-Glucosidase) activity [20].

### Molecular genetics investigations

#### DNA extraction and purification

DNA was isolated from whole blood using the standard salting-out method and quantified using a QIAxpert (Cat. No: 9002340) from Qiagen [21]. The DNA samples were purified using The Genomic DNA Clean & Concentrator™-25 (DCC™) Kit, from Zymo Research, Irvine, California, U.S.A (Cat. No. D4064) and were stored at − 20 °C until investigated.

#### Primary screening of the common Gaucher mutations (Leu444Pro)

The DNA samples were amplified in Thermal Cycler-2720 (Applied Biosystems, Inc. India) using our earlier described protocol [18]. The polymerase chain reaction (PCR) product was then subjected to restriction digestion by Restriction endonucleases *MspI* (New England Biolabs). In brief, 10 μl of PCR product was incubated with 0.5 μL of *MspI* (10 U/μl) at 37 °C for 3 h. The digested DNA fragments were separated on 2.5% agarose gel. The above protocol also covers the mutation Arg463Cys which can be distinguished from Leu444Pro on an agarose gel based on the different sizes of the DNA fragments. The codon numbering of the mutations described in this study uses the traditional amino acid residue numbering, which excludes the first 39 amino acids of the leader sequence used in the current nomenclature for *GBA* mutations.

### Single-gene sequencing (*GBA* gene)

*GBA* gene containing 11 exons was amplified using primer sets listed in the Additional file 1. The primers were standardized using nested PCR for specific amplification of the functional gene. For exon 1–2, 35 cycles of amplification; each consisting of initial denaturation (94 °C; 4 min), denaturation (94 °C; 30 s), annealing (65.5 °C; 30 s), elongation (72 °C; 30 s), and final elongation (72 °C; 10 min) were run. Amplification for exon 3–4 involved initial denaturation (96 °C; 2 min), denaturation (96 °C; 30 s), annealing (61 °C; 30 s), elongation (74 °C; 60 s), and final elongation (74 °C; 5 min) were run. Exon 5–11 included initial denaturation (96 °C; 2 min) followed by 33 cycles each consisting of denaturation (96 °C; 30 s), annealing (58 °C to 61 °C; 30 s), elongation (74 °C; 60 s), and final elongation (74 °C; 5 min). PCR products were run on the 2% agarose gel and visualized under ultraviolet transilluminator.

Sanger sequencing was performed by fluorescent dye-labeled genetic analysis system using capillary electrophoresis technology on the Applied Biosystems™ SeqStudio™ Genetic Analyzer with SeqStudio™ Data Collection Software. The PCR product, cleaned by Big Dye Terminator v3.1 Clean up, was further processed for cycle sequencing in Thermal Cycler-2720 considering the protocol of initial denaturation (96 °C; 1 min), denaturation (96 °C; 10 s), annealing (50 °C; 5 s), and elongation (60 °C; 4 min). Total 25 cycles were run. The cycle sequencing products (samples) were purified, resuspended in Hi-Di Formamide and transferred to 96 wells plate. The Samples, in a plate covered with septa, were denatured at 95 °C for 2 min and snap chilled at -20 °C for 2–3 min before proceeding with the sequencing. The sequences obtained were aligned to the available reference sequence (NM_001005741.2) in The National Center for Biotechnology Information (NCBI) GeneBank database to detect variation.

### In silico analysis

#### Prediction of the functional effect of the variants

Six in silico tools identifying the effect of DNA variants (MutationTaster2), coding non-synonymous variants (SIFT), coding and non-coding variants (FATHMM), and amino acid substitution (PolyPhen2, PROVEAN, and MutationAssessor) were employed.

### Homology modeling, structure validation and protein stability due to novel variants

The mutated protein structure of the novel variants was modeled using previously described protocol [22]. In brief, β-Glucosidase crystallographic structure (PDB ID: 1OGS) was used as the template and the Root Mean Square Deviation (RMSD) of the mutant structures with respect to the wild-type structure was calculated.

### Orthologous conservation of the *GBA* residues harboring the novel variant

The conservation of the *GBA* residues incorporating novel variants was checked across species according to the previously described protocol [22]. In brief, the protein sequence of *Homo sapiens* (NP_001005741) was aligned along with other species using Clastal Omega; an online multiple sequence alignment program.

### Population screening for c.1448T>C (Leu444Pro) variant by RFLP-PCR

Total 1200 unrelated and healthy population with no history of GD in the family or near relatives were enrolled for the study from different regions of India. The blood samples were collected after an ethics committee approval from the Institute and written informed consent. The DNA was isolated as described previously and was screened for the c.1448T>C (Leu444Pro) variant using previously described RFLP-PCR protocol. The sample size was planned by an online sample size calculator (http://www.raosoft.com/samplesize.html). Allele frequency was calculated through an online Hardy-Weinberg calculator (http://perinatology.com/calculators/Hardy-Weinberg.htm). Sanger sequencing, using the aforementioned primers, confirmed the results.

## Results

The patients included in the study presented unexplained hepatomegaly, moderate splenomegaly, anemia, and thrombocytopenia with or without any bone abnormality. Table 1 covers the clinical details and the demographic profile of the patients. Bone marrow aspirates in four cases showed the presence of classical Gaucher cells suggesting the possibility of GD. In addition to the above, patient P_1_ had difficulty in walking since 15 years of age and his bone X-ray revealed sclerosis and avascular necrosis of the left femur. Patient P_3_ had undergone splenectomy at the age of 17 years. The pelvis MRI report of the patient P_5_ revealed hyperintensities over the upper shaft of the right femur along with changes in pelvic floor muscles. Two patients (patient P_5_ and P_6_) were on imiglucerase Enzyme Replacement Therapy (ERT) before their molecular investigation. None of the patient manifested any neurological involvement. They had an uneventful childhood and the symptoms appeared in early adulthood.

**Table 1:** Clinical details and demographic profile of the adult patients with type I Gaucher disease

Patient ID	P_1_	P_2_	P_3_	P_5_	P_6_	P_7_
Age at the time of investigation (in years)	20	20	26	25	28	40
Sex	M	F	F	M	F	F
Region	GJ	GJ	GJ	PB	UP	MH
Symptoms						
Deep superficial reflexes					+	
Edema (Pelvis)				+		
Heaviness in abdomen				+		
Generalized weakness						+
Splenomegaly	+	+	+	+	+	+
Hepatomegaly	+		+	+		
Hematological abnormalities						
Anemia	+	+			+	+
Thrombocytopenia	+			+	+	+
Cytopenia	+					
Vacuolated lymphocytes					+	
Bone marrow analysis (presence of Gaucher cells)	+			+	+	+
Bone abnormalities						
Difficulty in walking	+					
Avascular necrosis (left femur)	+					
Enzyme Replacement Therapy				+	+	

*Abbreviation*: *F* Female, *GJ* Gujarat, *MH* Maharashtra, *M* Male, *PB* Punjab, *UP* Uttar Pradesh

Clinical history of the patient P_4_ is unavailable

### Biochemical analysis

Five patients (P_1_, P_2_, P_4_, P_5_, and P_7_) expressed a markedly elevated level of plasma chitotriosidase ranging from 1670 to 72,000 nmol/h/ml plasma. Patient P_6_ presented a normal level of plasma chitotriosidase (102.4 nmol/h/ml plasma) and undetectable chitotriosidase activity was noted in the patient P_3_. β-Glucosidase enzyme assay from leukocytes revealed a significantly reduced β-Glucosidase enzyme activity (≤ 10%) in all the patients (Table 2). This deficient activity confirmed the diagnosis of GD.

**Table 2:** Biochemical and molecular analysis of adult patients with type I Gaucher disease

Patient ID	Biochemical analysis		Molecular analysis			Allele Frequency		dbSNP reference sequence	Reference
	Plasma Chitotriosidase (nmol/hr/ml plasma)	β-Glucosidase (nmol/hr/mg protein)	Variant location (*GBA* gene)	Nucleotide change (Amino Acid change)	Zygosity	1000 Genomes	ExAC		
P_1_	54,503.7	2.5^#^	Exon 10	c.1448T>C (Leu444Pro)	Het	0.0034	0.0031	rs421016	[18]
			Exon 8	c.1102C>T (Arg329Cys)	Het	NR	0.00002472	rs374306700	[18]
P_2_	72,000.0	1.2^#^	Exon 10	c.1459G>A (Ala448Thr)	Hom	NR	NR	rs878853317	In this study
P_3_	0.0	1.2^#^	Exon 10	c.1459G>A (Ala448Thr)	Hom	NR	NR	rs878853317	In this study
P_4_	14,378.0	0.24^#^	Exon 8	c.1060G>A (Asp315Asn)	Hom	NR	0.000008243	rs398123526	[24]
P_5_	1670.0	3.5^**†**^	Exon 10	c.1448T>C (Leu444Pro)	Het	0.0034	0.0031	rs421016	[18]
			Exon 3	c.167T>G (Val17Gly)	Het	NR	NR	rs878853318	In this study
P_6_	102.4	4.65^†^	Exon 10	c.1459G>A (Ala448Thr)	Het	NR	NR	rs878853317	In this study
			Exon 5	c.492C>G (Ser125Arg)	Het	NR	0.00003295	–	[24]
P_7_	54,503.7	1.5^#^	Exon 9	c.1300C>T (Arg395Cys)	Hom	NR	NR	–	[18]

*Abbreviations*: The Single Nucleotide Polymorphism database (dbSNP), The Exome Aggregation Consortium (ExAC), Heterozygous (Het), Homozygous (Hom), Not Reported (NR)

Plasma Chitotriosidase normal range: 28.66–62.94 nmol/hr/ml plasma

^#^β-Glucosidase enzyme activity done at our center (normal range: 4.0–32.0 nmol/hr/mg protein)

^†^β-Glucosidase enzyme activity done at other centers (normal range: 10.0–45.0 nmol/hr/mg protein)

The above variants refers to the *GBA* gene with transcript ID ENST00000327247.5 and reference sequence number NM_001005741.2

### Molecular analysis

**Fig. 1:**
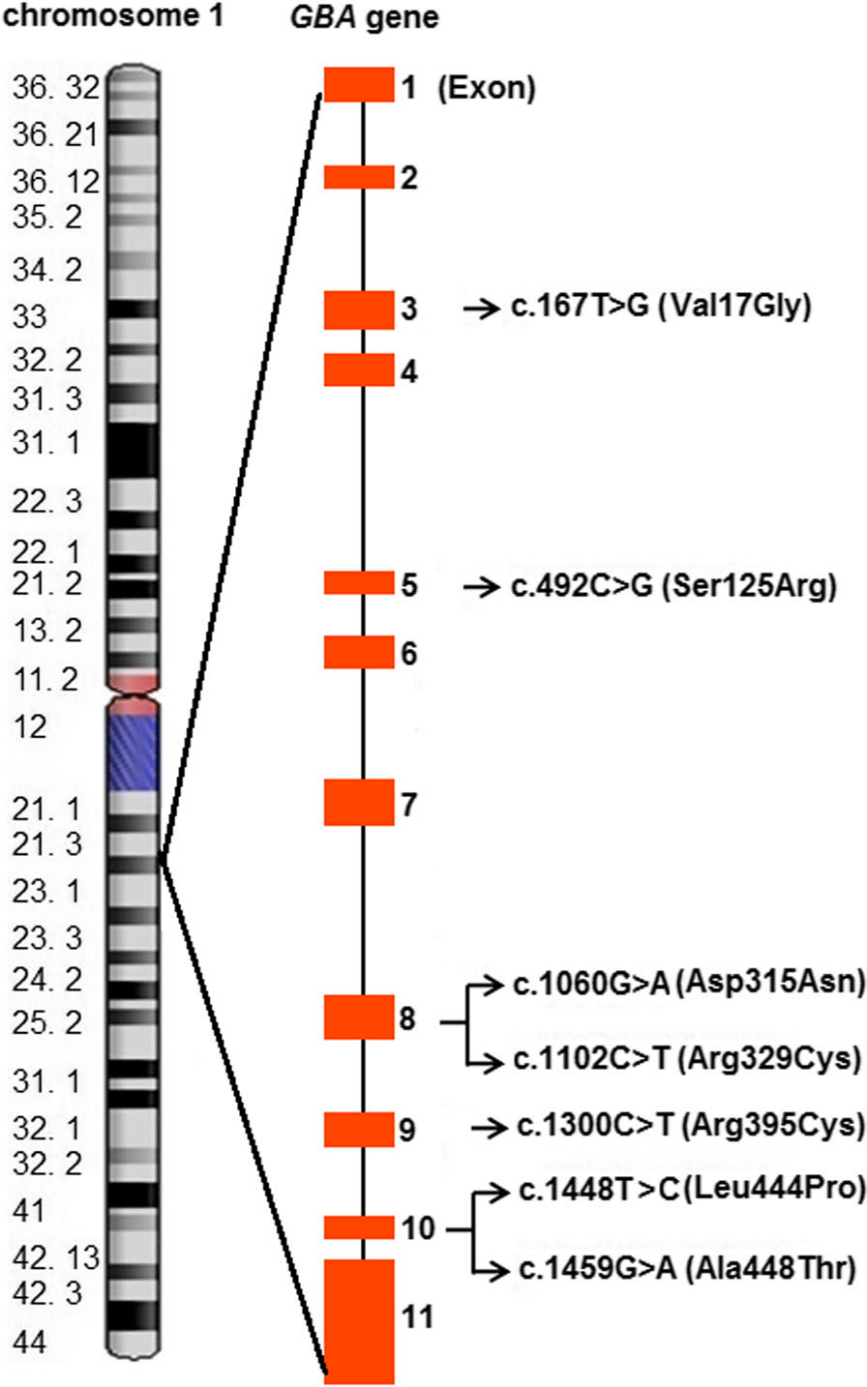
Illustrative representation of the distributions of the variants identified in Indian adult Gaucher patients investigated in this study. Variations in exon 3, 5, 8, 9, and 10 of *GBA* gene were observed

The identification of genetic cause of GD in all the patients involved an initial screening for the common mutations (Leu444Pro) observed in GD [18, 23]. Patient P_1_ and P_5_ were found to carry a heterozygous copy of c.1448T>C (Leu444Pro) variant in exon 10 of *GBA* gene. As a single variant or no variant was detected in the initial screening, the further investigation involved the sequencing of the complete coding region of *GBA* gene in all the patients. Sanger sequencing identified four patients (P_2_, P_3_, P_4_, and P_7_) with a homozygous variation and three patients (P_1_, P_5_, and P_6_) with compound heterozygous variation in *GBA* gene. Figure 1 depicts an illustrative representation of the location of the variants identified through Sanger Sequencing.

The variants identified as compound heterozygous in the patient P_1_ are known pathogenic variants and have also been identified in our previously published data [18]. The patients P_2_ and P_3_ harbor a homozygous copy of a variant c.1459G>A (Ala448Thr) in exon 10 (Table 2). This mutant allele is not reported in the 1000 Genomes database or The Exome Aggregation Consortium (ExAC). To the best of our knowledge, this variant has not been previously reported in the patients with GD. The patient P_4_ carried a known variant; previously reported in GD [24]. The patient P_5_ involved a c.167T>G (Val17Gly) variant along with known p.Leu444Pro variant in a compound heterozygous form. The variant p.Val17Gly is not reported in either 1000 Genomes or ExAC databases. Also, the variant has not been previously established with GD.

Patient P_6_ was identified as compound heterozygous with a variant Ala448Thr (as identified in the patient P_2_ and P_3_) and another known reported variant Ser125Arg [24]. The patient P_7_ harbors a known variant previously reported by us [18].

**Fig. 2:**
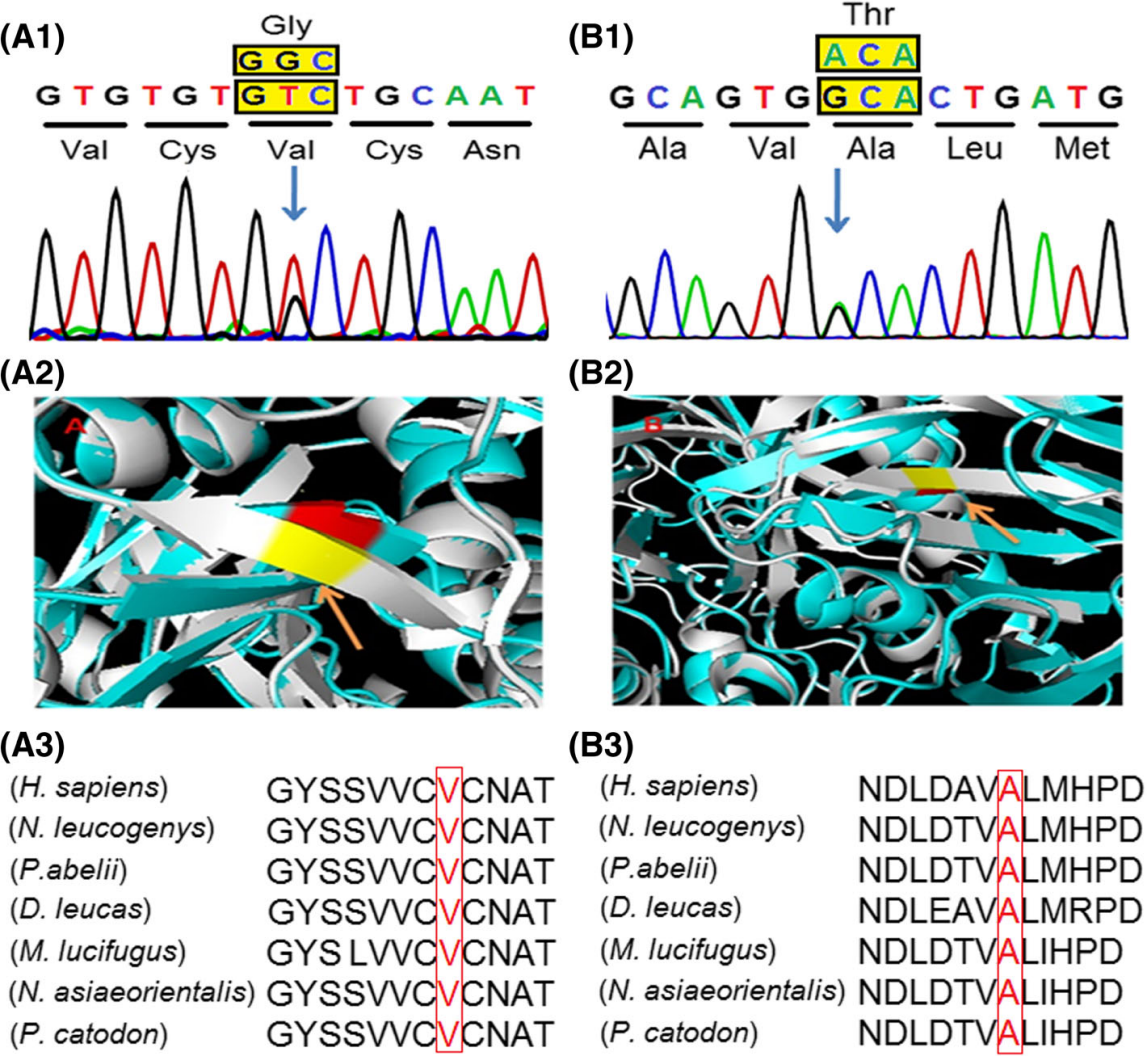
Identification of novel variants. (A1) and (B1): Sanger sequencing revealed two novel variants Val17Gly and Ala448Thr in *GBA* gene. (A2) and (B2): The superimposed model of native structure (blue) and mutant structure (white) of Val17Gly and Ala448Thr shows conformational changes in the β pleated sheet; indicated by an arrow. (A3) and (B3): the multiple alignments of the protein sequence region surrounding the variants Val17Gly and Ala448Thr against various orthologous sequences. The conserved residues Valine (V) and Alanine (A) in the orthologs are mark red

The in silico tools described above established the functional effects of the variants identified [see Additional file 2]. The novel variants (Ala448Thr and Val17Gly) were found to be disease-causing. Protein homology modeling further established the severe damaging effect of these novel variants. The variation occurred at highly evolutionarily conserved and functionally active residual domain in the protein leading to conformational changes or destabilization of the protein structure as shown in Fig. 2. The RMSD values for the modeled mutants were significant for the pathogenicity of the novel variants (0.147 for p.Ala448Thr and 0.144 for p.Val17Gly) compared to ~ 0.6 Å RMSD for wild type protein structure [25].

### Population screening for c.1448T>C (Leu444Pro) common mutation

The study included 17 subjects from the Eastern zone, 740 subjects from the Western zone, 114 subject from the Northern zone, 165 subjects from the Southern zone, and 164 subjects from the central zone of India. The sample size of 1200 subjects yielded a 3.6% margin of error with a confidence limit of 99%. From 1200 subjects screened, 2 subjects exhibited a carrier status for c.1448T>C (Leu444Pro) as identified on the agarose gel. Sanger sequencing confirmed their heterozygosity [see Additional file 3]. This has yielded the carrier frequency of 1:600 for the p.Leu444Pro variant in *GBA* gene. Using the Hardy-Weinberg formula, the allele frequency of wild-type allele (T) is found to be 0.97113 and that of a mutant allele (C) is 0.02887 (Table 3).

**Table 3:** Carrier frequency analysis of Gaucher disease common mutation c.1448T>C (Leu444Pro)

Disease	Gaucher
Gene	*GBA*
Mutation	c.1448T>C
Samples tested	1200
Heterozygote detected in this study	T/C: 2
Carrier frequency	1:600
allele frequency	(T>C) 1:1200
Wild-type allele frequency	T: 0.97113
Mutant allele frequency	C: 0.02887
Transcript identification	ENST00000327247.5
Mutation type	Missense
Mutation effect	Glucocerebrosidase enzyme deficiency
Promising therapeutic approaches	Enzyme Replacement Therapy (ERT)

## Discussion

Patients enrolled in this study exhibited clinical presentations like splenomegaly in all, with visceral infiltration (pancytopenia), and bone manifestations in some of them. Bone marrow examination in the majority of the patients showed classical Gaucher cells. Gaucher cells mainly infiltrate liver, spleen, bone marrow and lungs. All the patients in our study manifested unexplained splenomegaly, which is most commonly observed in type 1 GD with its prevalence in more than 90% of cases; however, hepatomegaly is less frequent with its estimate observation in 60–80% patients [14].

Bone marrow aspirates show the presence of Gaucher cells which are lipid encroached macrophages. Activated macrophages produced a chitotriosidase enzyme (EC 3.2.1.14) which makes it an apt diagnostic biomarker for the evaluation of GD patients [26]. The present study showed markedly elevated levels of plasma chitotriosidase enzyme activity in five out of seven patients (~ 71%). However, low or undetectable chitotriosidase enzyme activity in another two patients might possibly due to the presence of a null allele in Chitinase 1 (*CHIT1*) gene [27]. The comparatively low chitotriosidase activity in patients P_5_ and P_6_ might also be attributable to the enzymatic replacement therapy. A study by Hollak et al. reported a dramatic decrease in the chitotriosidase activity in the patients subjected to enzyme supplementation therapy [28].

Three mutations Arg329Cys, Arg395Cys, and Ser125Arg identified in the patients are known to be localized in Domain II/TIM Barrel which contains the catalytic active site of the protein and hence affects the catalytic function by producing mild to severe phenotype [29]. This may justify the occurrence of heterogeneous phenotype in these patients (from hepatosplenomegaly, thrombocytopenia to presence or absence of bone involvement).

Moreover, two novel mutations (Ala448Thr and Val17Gly) have been detected in the present series without any bone involvement. The in silico protein modeling analysis has demonstrated that mutant allele Ala448Thr is located in Ig like domain (domain II) at a significant distance from the active site and alters folding of this domain hence disrupting the hydrophobic core. Another allele Val17Gly is located in domain I of the GBA molecule which leads to misfolding of the protein due to alteration in the β-sheet.

One of the patients in the present study harbors a compound heterozygous mutation (Leu444Pro/ Arg329Cys) along with a phenotype of avascular necrosis as the primary sign with mild hepatosplenomegaly while another patient with Leu444Pro/Ser125Arg mutation present with thrombocytopenia and splenomegaly. Very few adult patients are reported as compound heterozygous for Leu444Pro and second rare allele (Leu444Pro/rare allele) [10, 11, 30]. Ito et al., 2013 reported a compound heterozygous mutation Leu444Pro/Asp409His in a 21-year-old adult male with GD associated with temporal intestinal hemorrhage [31]. The gene conversion and crossing over events between *GBA* gene and its highly homologous pseudogene (ps*GBA)* produces mutated complex alleles, which are frequently, associated with the mutations Asn370Ser and Leu444Pro. A study by Saleem et al. reported four Egyptian type 1 GD patients with compound heterozygous Asn370Ser/RecNciI [32]. A study by Lee et al. reported a case of Indonesian Chinese boy harboring compound heterozygous Leu444Pro/RecNclil [33]. Similar study in Ukrainian patients, by Olkhovych et al., revealed four patients of type I GD with compound heterozygous Leu444Pro and complex mutants [34]. Though Asn370Ser is not seen in Indian population, our recent unpublished work on 100 GD children has observed Leu444Pro with complex allele. Few studies have demonstrated a strong link between late-onset Parkinson disease (LOPD) and presence of the Leu444Pro allele [35, 36]. This warrants the follow-up of our patients for LOPD. In addition to this, type 1 GD and/or adult GD is also found to be associated with myoclonic epilepsy, malignant bone tumor, synucleinopathies including Parkinson’s disease and dementia with Lewy bodies [37–40].

Endogamous marriage practice in India has increased the burden of several autosomal recessive diseases within each specific community. The cost of therapeutic approaches like ERT, small molecular chaperones, and low success with Stem Cell Transplantation limits their availability to wide population [41]. Therefore, the only means of preventing the occurrence of these diseases is either by prenatal diagnosis or pre-marriage counseling. Our earlier studies have identified GD as the most common storage disorder with Leu444Pro as the most common mutant allele in an Indian population [4, 18]. A study by Kadali et al. also established a higher incidence of GD in India [26]. The present study identified 2 unrelated subjects with no family history of GD to be heterozygous for c.1448T>C (Leu444Pro) mutation suggesting the carrier frequency to be 1:600. Considering a heterogeneous population in India with various social, regional and community-based marriage practices, it is very expected that certain area in the country is likely to have higher incidences of GD where consanguineous marriage practice is common. Our previous study revealed that regions of Maharashtra have a higher rate of GD as compared to other parts of the country [4].

## Conclusions

The given study reports seven patients with an adult onset of type 1 GD with all mutations clustered in exon 8 and 10. Also, the study contributes two novel variants to the spectrum of *GBA* gene mutations along with providing knowledge about the prevalence of most common *GBA* gene mutation c.1448T>C (Leu444Pro) mutation in a general Indian population. This can help in maintaining population health quotient by doing pre-marital counseling, especially in endogamy community and hence minimizing the disease burden amongst the population. In view of milder clinical manifestation in the course of adult GD, differential diagnosis of GD needs to be suspected in patients with unexplained anemia, pancytopenia and/or hepato/splenomegaly.

## Additional files

Additional file 1: List of primers used for *GBA* gene sequencing. The exons and the exon-intron boundaries of the GBA gene were bidirectionally sequenced using the given set of primers. (DOC 30 kb)

Additional file 2: In silico analysis of the functional effect of the variants identified in the adult patients with type 1 GD. The in silico tools predicting the effect of DNA variants, coding non-synonymous variants, amino acid substitution, and non-coding variants were employed to predict the functional effect of the variants identified in the given study. (DOC 37 kb)

Additional file 3: Population screening of the c.1448T>C (Leu444Pro) variant. The screening identified two carriers of Leu444Pro out of 1200 population. This gives the carrier frequency of 1:600. Sanger sequencing confirmed the results. (DOC 236 kb)

Additional file 4: ClinVar Accession ID of the variants generated in the given study. The variants identified through Sanger sequencing are reported in NCBI ClinVar database. The file provides accession ID and the links to an individual variant. (DOC 29 kb)

## Abbreviations

CHIT1: Chitinase 1
ERT: Enzyme Replacement Therapy
ExAC: The Exome Aggregation Consortium
FATHMM: Functional Analysis Through Hidden Markov Models
GBA: Glucosylceramidase beta gene
GD: Gaucher disease
LOPD: Late-onset Parkinson disease
NCBI: The National Center for Biotechnology Information
PolyPhen2: Polymorphism Phenotyping v2
PROVEAN: Protein Variation Effect Analyzer
RMSD: Root Mean Square Deviation
SIFT: Scale-invariant feature transform
