# Supplementary material for: Biochemical and molecular characterization of adult patients with type I Gaucher disease and carrier frequency analysis of Leu444Pro - a common Gaucher disease mutation in India
Source: BMC Med Genet. 2018 Oct 1;19:178. doi: 10.1186/s12881-018-0687-5 (PMC6167838; doi:10.1186/s12881-018-0687-5)
Supplement: Supplementary file 1 — List of primers used for GBA gene sequencing. The exons and the exon-intron boundaries of the GBA gene were bidirectionally sequenced using the given set of primers. (DOC 30 kb) [file 12881_2018_687_MOESM1_ESM.doc]

**Primer sets for *GBA* gene Sanger sequencing**

| **Location** | **Primers** | |
| --- | --- | --- |
|  | **Sense 5'<---->3'** | **Antisense 3'<---->5'** |
| Exon 1 | CCTAAAGTTGTCACCCATAC | CAACCCTTCTGATGACAACT |
| Exon 2 | GGAGAGGGGCTTGCTTTTCA | GGAGGCAGAGGTTGGAATGA |
| Exon 3-4 | CAAGGGGTGAGGAATTTTGA | CACCACTGCACTCCTGTCTC |
| Exon 5-6 | TGGCCCTGACTCAGACACTA | CTGATGGAGTGGGCAAGATT |
| Exon 7 | GGCTGTTCTCGAACTCCTGA | ATAGTTGGGTAGAGAAATCG |
| Exon 8 | AGTTGCATTCTTCCCGTCAC | ATCATGGTTCCCCAGAGTTG |
| Exon 9 | CAGCTGCCTCTCCCACAT | GTGTGCCTCTTCCGAGGTT |
| Exon 10-11 | GAGAGCCAGGGCAGAGCCTC | CTCTTTAGTCACAGACAGCG |
