# Supplementary material for: Biochemical and molecular characterization of adult patients with type I Gaucher disease and carrier frequency analysis of Leu444Pro - a common Gaucher disease mutation in India
Source: BMC Med Genet. 2018 Oct 1;19:178. doi: 10.1186/s12881-018-0687-5 (PMC6167838; doi:10.1186/s12881-018-0687-5)
Supplement: Supplementary file 2 — In silico analysis of the functional effect of the variants identified in the adult patients with type 1 GD. The in silico tools predicting the effect of DNA variants, coding non-synonymous variants, amino acid substitution, and non-coding variants were employed to predict the functional effect of the variants identified in the given study. (DOC 37 kb) [file 12881_2018_687_MOESM2_ESM.doc]

***In silico* prediction of the functional effect of the variants identified in the adult patients with type 1 GD**

| **Patient ID** | **cDNA position**  **(Amino Acid change)** | **MutationTaster2** | **SIFT** | **FATHMM** | **PolyPhen-2** | **PROVEAN** | **Mutation Assessor^†^** |
| --- | --- | --- | --- | --- | --- | --- | --- |
| **P_1_** | c.1448T>C  (Leu444Pro) | Disease causing | Damaging | Pathogenic | Possibly damaging | Deleterious | Medium |
|  | c.1102C>T  (Arg329Cys) | Disease causing | Damaging | Pathogenic | Possibly damaging | Deleterious | Medium |
| **P_2_** | c.1459G>A (Ala448Thr) | Disease causing | Damaging | Pathogenic | Possibly damaging | Deleterious | Medium |
| **P_3_** | c.1459G>A (Ala448Thr) | Disease causing | Damaging | Pathogenic | Possibly damaging | Deleterious | Medium |
| **P_4_** | c.1060G>A (Asp315Asn) | Disease causing | Tolerated | Pathogenic | Possibly damaging | Deleterious | Low |
| **P_5_** | c.1448T>C (Leu444Pro) | Disease causing | Damaging | Pathogenic | Possibly damaging | Deleterious | Medium |
|  | c.167T>G (Val17Gly) | Disease causing | Damaging | Pathogenic | Possibly damaging | Deleterious | Medium |
| **P_6_** | c.1459G>A (Ala448Thr) | Disease causing | Damaging | Pathogenic | Possibly damaging | Deleterious | Medium |
|  | c.492C>G (Ser125Arg) | Disease causing | Damaging | Pathogenic | Possibly damaging | Deleterious | High |
| **P_7_** | c.1300C>T (Arg395Cys) | Disease causing | Damaging | Pathogenic | Possibly damaging | Deleterious | Low |

Abbreviations: The Functional Analysis Through Hidden Markov Models (FATHMM), Polymorphism Phenotyping version 2 (PolyPhen-2), Protein Variation Effect Analyzer (PROVEAN), The Sorting Intolerant from Tolerant (SIFT)

† Impact of amino acid substitution on protein function
