# Supplementary material for: Biochemical and molecular characterization of adult patients with type I Gaucher disease and carrier frequency analysis of Leu444Pro - a common Gaucher disease mutation in India
Source: BMC Med Genet. 2018 Oct 1;19:178. doi: 10.1186/s12881-018-0687-5 (PMC6167838; doi:10.1186/s12881-018-0687-5)
Supplement: Supplementary file 3 — Population screening of the c.1448T>C (Leu444Pro) variant. The screening identified two carriers of Leu444Pro out of 1200 population. This gives the carrier frequency of 1:600. Sanger sequencing confirmed the results. (DOC 236 kb) [file 12881_2018_687_MOESM3_ESM.doc]

**Population Screening for c.1448T>C (Leu444Pro) common mutation**


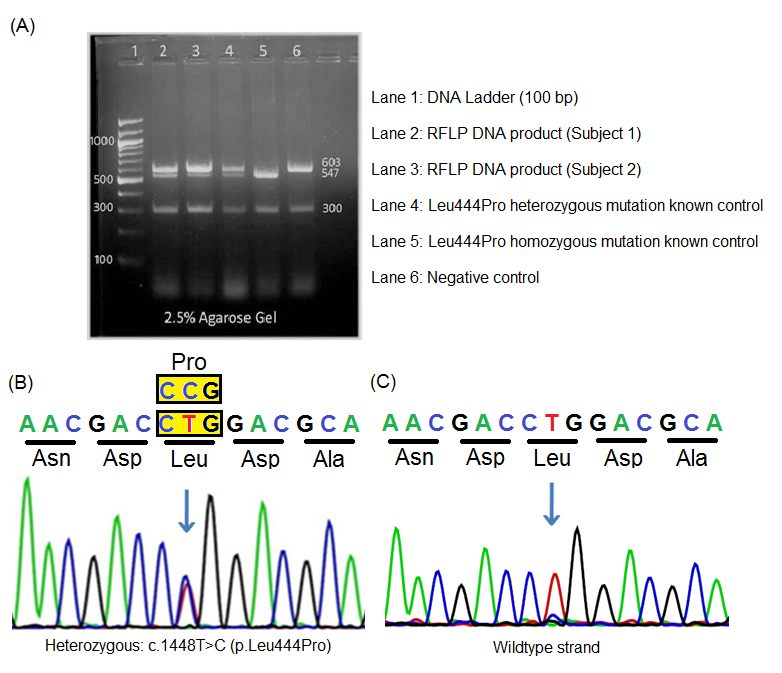


**Figure:** Restriction Fragment Length Polymorphism (RFLP): (A) Screening 1200 unrelated and healthy individuals for (c.1448T>C) Leu444Pro mutation in the *GBA* gene identified 2 subjects as carriers. The rest 1198 subjects were found normal for the said mutation. This has yielded the carrier frequency of 1:600 for the Leu444Pro mutant allele of *GBA* gene. Sanger sequencing: (B) And (C) Sanger sequencing confirmed the results established through PCR-based techniques.
