# Supplementary material for: Biochemical and molecular characterization of adult patients with type I Gaucher disease and carrier frequency analysis of Leu444Pro - a common Gaucher disease mutation in India
Source: BMC Med Genet. 2018 Oct 1;19:178. doi: 10.1186/s12881-018-0687-5 (PMC6167838; doi:10.1186/s12881-018-0687-5)
Supplement: Supplementary file 4 — ClinVar Accession ID of the variants generated in the given study. The variants identified through Sanger sequencing are reported in NCBI ClinVar database. The file provides accession ID and the links to an individual variant. (DOC 29 kb) [file 12881_2018_687_MOESM4_ESM.doc]

The dataset generated and/or analyzed during the current study is available in the NCBI ClinVar repository. Following are the accession ID of the variants submitted to the ClinVar repository

1. **Variant c.1459G>A (Ala448Thr) in exon 10 of *GBA* gene. (Identified in the patient P_2_)**

ClinVar Accession ID: SCV000282508.1

[<https://www.ncbi.nlm.nih.gov/clinvar/variation/236402/>]

1. **Variant in c.1448T>C (Leu444Pro) exon 10 of *GBA* gene. (Identified in the patient P_5_)**

ClinVar Accession ID: SCV000282509.2

[<https://www.ncbi.nlm.nih.gov/clinvar/variation/424819/>]

1. **Variant c.167T>G (Val17Gly) in exon 3 of *GBA* gene. (Identified in the patient P_5_)**

ClinVar Accession ID: SCV000282509.2

[<https://www.ncbi.nlm.nih.gov/clinvar/variation/424819/>]
